# Soil Quality and Trace Element Risk in Urban and Rural Kitchen Gardens: A Comparative Analysis

**DOI:** 10.3390/toxics13080697

**Published:** 2025-08-20

**Authors:** Diego Arán, Osvaldo Santos, Rodrigo Feteira-Santos, Yacine Benhalima, Erika S. Santos

**Affiliations:** 1 LEAF—Linking Landscape, Environment, Agriculture and Food Research Center, Instituto Superior de Agronomia, Universidade de Lisboa, Tapada da Ajuda, 1349-017 Lisbon, Portugal; yacinebenhalima@isa.ulisboa.pt (Y.B.); erikasantos@isa.ulisboa.pt (E.S.S.); 2 Associate Laboratory TERRA, Instituto Superior de Agronomia, Universidade de Lisboa, Tapada da Ajuda, 1349-017 Lisbon, Portugal; osantos@medicina.ulisboa.pt (O.S.);; 3 Institute of Environmental Health, Lisbon School of Medicine, Universidade de Lisboa, Av. Prof. Egas Moniz, 1649-028 Lisbon, Portugal

**Keywords:** pollution index, enrichment factor, PLI, potential ecological risk, community gardens, allotment gardens, urban soils

## Abstract

The development and use of urban spaces for food production is increasing in response to the search for healthier foods and contact with nature. These spaces can be created or built on materials of various types, which might contain potentially toxic elements (PTEs). This study focuses on the evaluation of soil fertility and contamination levels in urban and rural kitchen gardens in Lisbon, Portugal. Soils of twenty kitchen gardens (n_urban_ = 15; n_rural_ = 5) were sampled, and their physicochemical characteristics and the contents of PTEs in the total and available fractions were analyzed. The results were used to calculate contamination indices and associated ecological risk. The soils of the urban and rural kitchen gardens had a neutral pH, with the presence of carbonate forms, and moderate-to-high organic matter contents, although with a clear nutritional imbalance. Regarding PTEs, both urban and rural kitchen gardens soils showed elevated levels of certain elements (e.g., Cr, Ni, Cu), exceeding the maximum allowable values established by Portuguese regulations. However, the available fraction of these elements was generally low. Contamination indices ranged from mild to considerable in isolated cases, with no general multi-element contamination or ecological risk. This suggests that associated environmental and health risks are minimal, although periodic monitoring of kitchen gardens’ soil quality is necessary to ensure and maximize the health benefits.

## 1. Introduction

Urbanization is a growing, worldwide phenomenon. The United Nations (UN) has estimated that the urban population will increase from 30% in 1950 to 68% of total population in 2050, a proportion even higher in more developed countries (86%) [1]. Movements and concentration of population in urban areas have positively affected societies’ wellbeing and public health, by improving their residents’ living standards [2,3], associated with more opportunities or access to healthcare, for instance by providing more opportunities and better access to healthcare.

However, urban living and exposure to urban environment-associated risk factors can also be harmful to health and mortality [3,4], posing health challenges to individuals related to the urban environment and lifestyle. Estimations of mortality attributable to urban environmental risks highlight a negative impact, for instance, from living exposed to excessive air pollution [5,6], extreme heat [7] and insufficient green spaces [8,9].

Green infrastructure in cities and urban gardening is particularly important, preserving health and wellbeing of urban populations and offering recreational opportunities, physical activity and social cohesion [10,11,12,13,14]. The positive effects on health and wellbeing, especially mental health, of being in contact with natural environments within the urban setting have also been documented [15,16]. Despite heterogeneity across study designs and populations, findings seem to agree on the fact that exposure to green spaces has benefits for mental health outcomes [17,18,19,20]. Human–nature interactions in cities also provide opportunities for people to develop pro-environmental attitudes toward the environment, helping to increase their awareness of environmental issues [13,21].

Reconnecting urban populations with nature and agriculture has also been highlighted in the dimension of food systems serving cities. With many people already living in metropolitan areas and many more coming from rural areas, 80% of all food produced is expected to be consumed in cities by 2050 [22]. However, food supply chains for cities are becoming increasingly longer, often starting far from cities in order to deliver enough nutritious and safe food to urban populations [23]. In some cases, urbanization has led to phenomena known as food deserts and food swamps, which are geographic areas characterized by either limited access to diverse, fresh or nutritious foods or to an overabundance of high-energy and low-nutritional density foods, respectively [23]. As a result, some population subgroups are at higher risk of poor health outcomes, since limited access to nutritious, safe and affordable food is limited. Many times, paradoxically, malnutrition can manifest in multiple forms, and an association has been observed between food insecurity and obesity [24,25,26].

In accordance with the Food and Agriculture Organization framework for the Urban Food Agenda, urban food policy should focus on addressing food insecurity and building resilience in urban food systems, for instance, by promoting rural–urban linkages and leveraging sustainable food production in or near cities [27]. Different strategies have thus been proposed to shrink the gap between where food is produced and where it is consumed, namely urban agriculture and in-city farming, with benefits in different domains, such as promoting local supply chains, improving wellbeing and access to food and hence food security, as well as other positive impacts for biodiversity and climate resilience [28]. Moreover, urban gardening can have multiple positive outcomes, including physical, psychological and social benefits, which have been often described as being beneficial for human health in urban areas [11,12].

Nevertheless, urban gardens also have environmental risks which can harm human health. Soil contamination by potentially toxic elements (e.g., Pb, Cd, Cr, Ni, Zn) is one of the main concerns since these often accumulate in urban soils, due to long-term deposition from vehicle traffic, industrial emissions, and multiple other sources. Human exposure (with several possible transmission pathways) to these soil contaminants can occur through ingestion of home-grown produce, inhalation of resuspended soil particles, or direct contact with soil during gardening. Several studies have found PTE concentrations in urban garden soils often at toxic levels, posing potential risks for human health, namely for more vulnerable groups [29]. To address this issue, we evaluated soil fertility and PTE contamination in kitchen gardens (also designated as community gardens or allotment gardens) across urban Lisbon, Portugal, using rural kitchen gardens as a baseline for comparison. Our study addresses a critical knowledge gap regarding PTE contamination in Lisbon’s urban kitchen gardens. It contributes to clarifying the extent of PTE enrichment in these soils and associated exposure risks for local gardeners, providing evidence to inform public health guidelines, urban food policies, and environmental planning decisions for safer and more sustainable urban gardening.

## 2. Materials and Methods

### 2.1. Characterization of Study Area and Sampling Sites

The Lisbon Metropolitan Area is one of the most dynamic and urbanized regions in Portugal. The northern zone includes nine municipalities located north of Lisbon and the Tagus River. These municipalities combine urban and suburban areas with high population density, heavy traffic and industrial centers.

The climate is mild Mediterranean, classified as Csb/Csa under the Köppen system [30], is characterized by warm–hot and dry summers, while winters are mild and rainy. The proximity of the Atlantic Ocean attenuates the temperatures, particularly in coastal areas. The average annual rainfall is between 600 and 1000 mm, particularly between October and January (climatological normal for the Lisbon station, 1981–2010 and 1991–2020; [30]). The average annual temperature ranges from 11 to 24 °C (climatological normal for the Lisbon station, 1981–2010 and 1991–2020; [30]).

**Figure 1:**
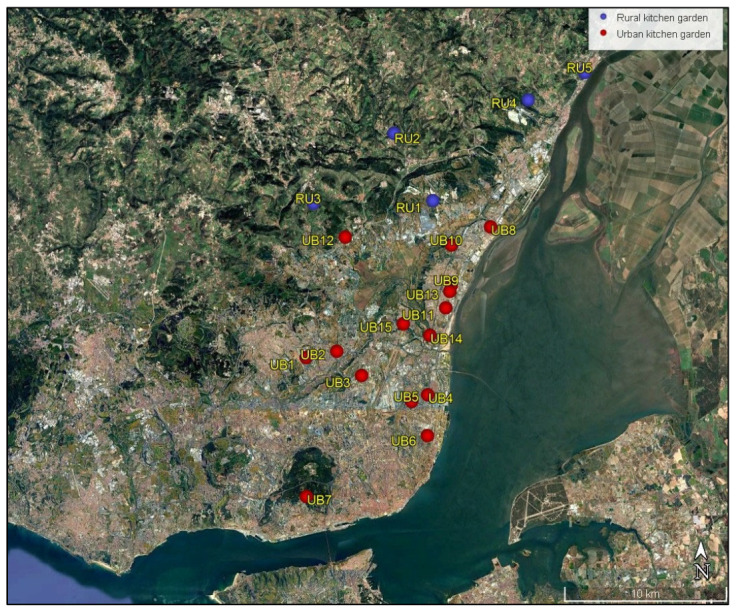
Location of soil sampling points in the urban kitchen gardens (red points; UB1–UB15) and rural kitchen gardens (blue points; RU1–RU5).

A total of 20 kitchen gardens were selected for the study: 15 in the northern zone of the Lisbon Metropolitan Area (N-LMA) and five in the adjacent rural zone, which served as a control group. The sampling areas were selected based on the presence of crops intended for human consumption, high levels of automotive traffic, and proximity (or not) to other urban sources of contamination. A homogeneous distribution was also ensured across the study area (Figure 1). In general, most of the urban kitchen gardens have a total area of 0.3 ha, divided into small plots between 10 and 20 m^2^. They are surrounded by households and intensive traffic flow.

The soils studied have a strong anthropic influence, with intensive agricultural use and continuous irrigation. The most representative crops vary, including lettuce, various varieties of tomato, beans, pumpkins or cabbages. The soils from urban kitchen gardens can be classified as Anthrosols or Technosols [31], depending on the origin of parent materials and amounts of artefacts, while soils from rural kitchen gardens are classified as Anthrosols [31].

### 2.2. Soil Sampling and Physico-Chemical Analysis

Soil sampling was carried out between spring and the beginning of summer in 2023. In each sampling area, a composite soil sample was collected until 20 cm of depth using plastic tools and then stored in labelled polyethylene bags. The composite soil sample was obtained by combining several trial points (total ≈ 3 kg) collected across the sampling area, to obtain a representative sample.

The soil samples were homogenized, and sieved at 2 mm. In a part of each sample, we determined the moisture content while the remaining sample was air-dried for subsequent physicochemical analysis. Standard methods for characterizing soil (dry fraction < 2 mm) were used: % fine and coarse fraction, pH and electrical conductivity in water (1:2.5 *m/V*), pH in KCl (1:2.5 *m/V*) [32], total N [33], total organic C [34], labile C by pyrophosphate extraction method [35], extractable P [36]; concentration of non-acid exchangeable cations extracted with NH_4_Cl 1M [37] and determined by Flame Atomic Absorption Spectroscopy (Thermo Scientific iCE™ 3300; Thermo Fisher Scientific, Waltham, MA, USA). Effective cation exchange capacity (CEC) was determined by summing the non-acid cations [38].

The multi-element concentration of the soil was determined for the following fractions: total (X-ray fluorescence spectrometry), pseudo-total and available. The element concentration in the pseudo-total fraction was determined in an international certified laboratory using the aqua regia method [39] and represents the amounts of elements associated with all soil phases except those associated with phyllosilicates. The available fraction was obtained as simulated leachates [40] and corresponds to the amounts of elements that can be taken up by plants and spread by the medium. The soil solutions from available fraction were analyzed by ICP-OES (Thermo Scientific iCAP 7000 series).

### 2.3. Evaluation of Environmental Risk and Contamination Level

Several indexes were calculated to evaluate the degree of soil contamination and the ecological risk to humans and the environment, based on the content of PTEs obtained.

The contamination factor (CF) is calculated as the ratio between the concentration of an element in the soil and its corresponding reference concentration (as shown in Equation (1)) [41,42,43]. As reference values, we considered the maximum limits proposed by Portuguese legislation for shallow soils and agricultural use [44].

CF = [PTE soil]/[PTE reference](1)

The soils are classified according to contamination levels as low contamination (CF < 1); moderate contamination (1 < CF < 3); considerable contamination (3 < CF < 6) and very high contamination (CF > 6) [42].

The degree of contamination (CD) considers the sum of the different contamination factors obtained for the PTE of a soil, described by Equation (2).

(2) $$\mathrm{CD}=\sum_{n}^{i}CF$$

The soils are classified as having a low degree of contamination (CD < 8); moderate degree of contamination (8 ≤ CD < 16), considerable degree of contamination (16 ≤ CD < 32), or very high degree of contamination (CD ≥ 32) [42].

The modified contamination factor (mCF) or degree of contamination, reported by Abrahim and Parker [45], Machender et al. [46] and Rahman et al. [47], includes the normalization of the degree of contamination by introducing the total number of PTEs considered into Equation (3).

(3) $$\mathrm{mCF}=\sum_{n}^{i}CF/n$$

This index classifies the quality of the soils as null to very low degree of contamination (mCF < 1.5); low (1.5 < mCF < 2); moderate (2 < mCF < 4); high (4 < mCF < 8); very high (8 < mCF < 16); extremely high (16 < mCF < 32); ultra-high (mCF > 32).

The pollution load index (PLI) measures the overall degree of contamination in an area or zone using Equation (4), determining areas with multi-element contamination with PLI > 1 [41,48].

(4) $$\mathrm{PLI}=\sqrt[{n}]{CF1\times CF2\ldots .CFn}$$

The ecological risk factor (ER) and the global potential ecological risk (GER), proposed by Hakanson [42], allow the evaluation of the possible ecological risk through the combination of the toxicity and concentration of each PTE, as well as the risk of a multi-element system. These indexes are calculated by multiplying the toxic response (Tr) of each element by its contamination factor according to Equations (5) and (6).

ER = TR × CF(5)

(6) $$\mathrm{GER}=\sum_{n}^{i}ER$$

The TR values for Zn, Cr, Cu, As, Cd, Hg, Ni, Co, Mo, Pb and Sb were 1, 2, 5, 10, 30, 40, 5, 5, 18, 5 and 13, respectively [42,49,50]. The risk classes were established by Hakanson [42] and Al-Dahar [51].

### 2.4. Statistical Analysis

Data from the studied soils were analyzed to evaluate the differences in soil characteristics between Groups 1 (urban kitchen gardens) and 2 (rural kitchen gardens). A non-parametric approach was adopted due to the small size of Group 2 and the significant differences between the groups, which could lead to deviations from the normality. For each parameter, the Mann–Whitney U test (equivalent to the Wilcoxon rank-sum test) was performed to compare the distributions between the two independent groups. This test does not assume normal distribution and is appropriate for ordinal or continuous data that do not meet parametric assumptions. The analysis was performed using the dplyr, readxl, and stats packages on R software version 4.4.3.

The Spearman correlation test was used to correlate the soil characteristics (r > 0.7; *p* < 0.01 and *p* < 0.05). Quality control of the analyses was performed using analytical replicates, certified standard solutions, reference materials (OREAS 45d, 922, 907, 263, 130, 521, 620, 610, 609b), blanks and laboratory standards (Actlabs and Laboratório de Pedologia, Instituto Superior de Agronomia—Universidade de Lisboa).

## 3. Results and Discussion

### 3.1. Soil Fertility and Multi-Elemental Content

The soils from urban kitchen gardens presented a dominance of the fine fraction and a neutral reaction condition (actual and potential, determined by pH in water and KCl, respectively) with low values of electrical conductivity (Table 1), being considered as non-saline soils. With respect to C forms, the inorganic C forms represented 47 ± 15% of the total C amount, indicating the presence of considerable carbonate forms that can maintain the current pH conditions. In the organic fraction, organic C contents are considered as high [52,53], although the more easily degraded labile forms represent on average 46% (determined by labile C/organic C ratio). This fact is consistent with the low C/N values calculated (8.11 ± 2.09). These conditions, which have a high level of labile C forms and low C/N ratios, contribute to the quick degradation of organic matter and availability of nutrients to the soil–plant system. However, there is usually also a significant loss of N [53]. In fact, total N contents were correlated with organic C (r = 0.96) but the values were low rounding 2.0 g/kg (Table 1). Soils collected in urban and green areas from Lisbon also presented values of pH, electrical conductivity and total N in same range [54,55]. However, according to the same authors, the organic matter concentration varied with urban soils [54,55]. The high concentrations of organic matter obtained in some of these soils can be attributed to the lack of soil mobilization in gardens and parks, as well as the varying depths at which soil is collected. In fact, more organic matter accumulates in the top few centimetres of soil, and no-till farming can minimize its degradation.

The available P represents between 10 and 20% of total P in the urban soils, with very high contents in almost all samples, except for UB3 and UB10, which presented 8.69 and 10.52 mg/kg, respectively. Although the available P contents are considered as high, they showed a higher variability, reaching values of up to 897 mg/kg in UB12. No relation was obtained between organic matter and available P so, this variability can be associated with source materials of the soils and fertilization application. The presence of high levels of available P can contribute to fertility problems associated with the formation of stable solid phases with micronutrients such as Fe, Mn, or Zn [56,57,58,59], thereby reducing the concentration of these elements to the plants.

The cation exchange capacity is considered an indirect indicator of fertility. The CEC was high [52] in the soils from urban kitchen gardens (Table 1), with the exchange complex dominated by Ca (88% of the total CEC). Soils collected in urban and green areas from Lisbon also presented values of CEC in the same range [55]. The simultaneous presence of high concentration of exchangeable Ca and inorganic C can suggest the application of liming amendments based on Ca-carbonates to the soils, which is a common corrective of the pH in the agriculture sector. These conditions of Ca saturation in exchangeable complexes led to limitations in Mg uptake by the plants, with an imbalance in the Ca/Mg ratio [53].

On the other hand, although the fine fraction dominates in the soils from rural kitchen gardens (Table 1), the coarse fraction tends to increase compared to urban garden soils, varying between 12% and 35% of total. The soils from rural kitchen gardens showed a neutral current and potential reaction under low-to-medium conductivity conditions, indicating that they are non-saline soils [52,53]. Nevertheless, the pH values of KCl in rural garden soils were more acid, indicating significant differences compared to urban garden soils (*p* < 0.05). This greater difference between the actual and potential reaction (pH in water and KCl) indicates the dominance of negative charges in the soils associated with organic matter, which in this group of rural garden soils had higher average contents than in urban gardens soils (Table 1).

Concentration of inorganic C forms in rural garden soils represented a smaller proportion of the total (~35%). The labile C/Organic C ratio also showed a high presence of easily degradable forms of organic matter, which tend to quicky decomposition and mineralization. These results also agree with low C/N ratios (<10). In the studied area, the organic amendments used are manure and commercial compost in rural areas, and commercial compost or compost made from kitchen waste in situ in urban areas. All these amendments have a rapid decomposition rate. Although this type of organic amendment provides immediate benefits to the soil–plant system by quickly supplying nutrients from the exchange complex of organic matter and the organic matter itself after its decomposition, the amount of organic matter is small over the medium term, requiring frequent application [60,61].

The total P in soils from rural kitchen gardens showed contents significantly higher compared to those in urban areas, although this difference was not obtained in the available fraction (Table 1). The available P results showed a high variability (Table 1), possibly associated with chemical fertilization. Values ranged from around 13 mg/kg for RU1–RU2 to 267–398 mg/kg for RU3–RU6.

Similarly to urban kitchen soils, the CEC of the soils studied in rural areas was high [52], with mean values of 65.34 cmolc/kg and a predominance of Ca in the exchange complex (Table 1). Although high, the Ca/Mg ratios presented values lower than 10 for samples RU3 to RU5, implying that the soils may have deficiencies in Mg availability [53].

**Figure 2:**
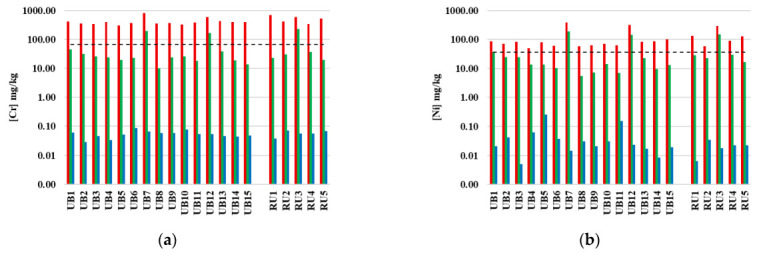

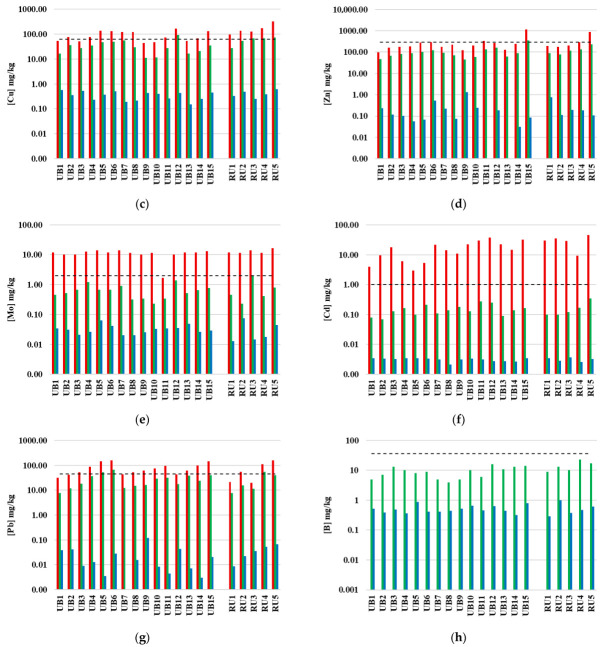
Distribution of total (red), pseudo-total (green) and available (blue) contents of (**a**) Cr; (**b**) Ni; (**c**) Cu, (**d**) Zn; (**e**) Mo; (**f**) Cd; (**g**) Pb and (**h**) B of soils collected from kitchen gardens in urban and rural areas of the northern Lisbon metropolitan area. The data are presented on a logarithmic scale. The black dashed line represents the value of Portuguese soil legislation for each element [44].

The usual soil risk assessment is based on comparing the pseudo-total concentrations of elements with the maximum values permitted by soil legislation. The concentrations of the 18 elements considered in Portuguese soil legislation [44] are presented in Table 2, grouped by soil from urban or rural kitchen gardens. These element concentrations were compared to the maximum allowed values (MAVs) established in this legislation for agriculture use. Furthermore, the contents of some representative elements (Cr, Ni, Cu, Zn, Mo, Cd, Pb and B) were determined by total, pseudo-total and available fractions. This allowed the associated risk of these elements to be assessed for each group and location (Figure 2).

The comparative evaluation of the pseudo-total content of the two soil groups showed significant differences only in the concentrations of Be and Cu, which reached the highest values in soils from rural kitchen gardens (Table 2). The contents of Be in all soils were low and below the MAV [44], while Cu contents in samples UB12, RU3, RU4, and RU5 exceeded MAV (Table 2; Figure 2).

Although the pseudo-total concentrations of other metals and metalloids in soils from the two established groups (urban or rural) were similar, the contents showed high heterogeneity, exceeding the MAV in some samples (46% of UB and 60% of RU) for V, Cr, Co, Ni, As, Mo, Ag, Sb, Pb and Hg (Table 2).

The pseudo-total content of V was high in some samples (UB7, UB12, RU1 and RU3), with values corresponding to double or triple those of the MAV [44]. However, these values were still within the range observed for different soils [62] suggesting that the concentrations in these samples can be associated with the local lithology. The presence of Sb above the MAV was only observed in urban kitchen garden soils (Table 2), especially in UB6 and UB15 samples. However, these values are within the ranges for basic rocks and below the phytotoxic limit [62]. The pseudo-total contents of Co exceeded MAV [44] in some samples (UB3, UB7, UB12, RU1 and RU3) with maximum values of 75.80 mg/kg in rural kitchen gardens (Table 2). The pseudo-total contents of As exceeded the MAV in the UB3 and RU4 samples, while the UB11 sample exceeded the MAV for Ag. However, Ag content of 2 mg/kg is considered the phytotoxic threshold [62].

The contents of Cr in the pseudo-total fraction exceeded the MAV by 2–3 times for samples UB7, UB12 and RU3, while the available fraction was less than 0.1 mg/kg in all samples (Figure 2). Although the soil availability of Cr is low, pH conditions would favor species associated with forms of Cr (VI), which are more soluble and toxic [63].

The pseudo-total content of Ni only exceeded the MAV in the sample UB7, which had a high concentration of 191 mg/kg (Figure 2). This is five times higher than the MAV and twice the average content of the lithosphere [63]. Nonetheless, phytotoxic concentrations for most plants and crops have been reported in the range of 40 to 260 mg/kg [62]. The available fraction of Ni in this soil sample (UB7) was low (0.01 mg/kg), similar to that in the other soils evaluated (Figure 2).

Elevated Pb concentrations in the pseudo-total fraction, greater than the MAV of 45 mg/kg, were identified in samples UB5, UB6 and RU4 (Figure 2). However, the availability of these elements is low, posing no direct risk. Sample UB5 also presented elevated pseudo-total contents of Hg.

The pseudo-total contents of B and Cd were below the MAV in all soils, indicating no risk (Table 2; Figure 2). The availability of B was low (Figure 2) corresponding to a mean value of 6.6% of pseudo-total concentrations in soils from urban kitchen gardens and 4.1% in rural gardens. B is considered the most mobile element of micronutrients but its availability in the studied soil is within normal ranges [62]. For Cd, its available fraction is lower than 0.01 mg/kg, representing between 0.9 and 5% of its pseudo-total fraction (Figure 2).

The results obtained in this study for the pseudo-total fraction, when compared to those obtained by Leitao et al. [64] for urban kitchen garden soils in Lisbon, showed similar contents of Be, Mo, Pb and Sb, higher concentrations for As, V, Cr, Cu, Ni and Zn, and the presence of additional elements such as Hg, Cd and Ag, which can pose some risk due to exceeding MAV at some sampling points. On the other hand, in soils collected in urban gardens or parks from Lisbon [54], pseudo-total concentrations of Cd were higher while Pb amount was much lower. Most of these soil samples presented concentrations of Cr and Ni in the same range [54]. Another study carried out in Lisbon’s urban and green areas also found pseudo-total concentrations of several elements within the same range [55].

**Figure 3:**
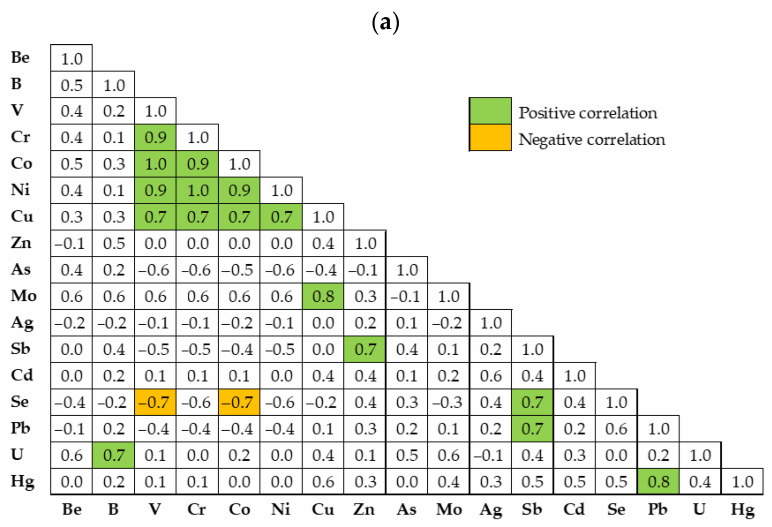

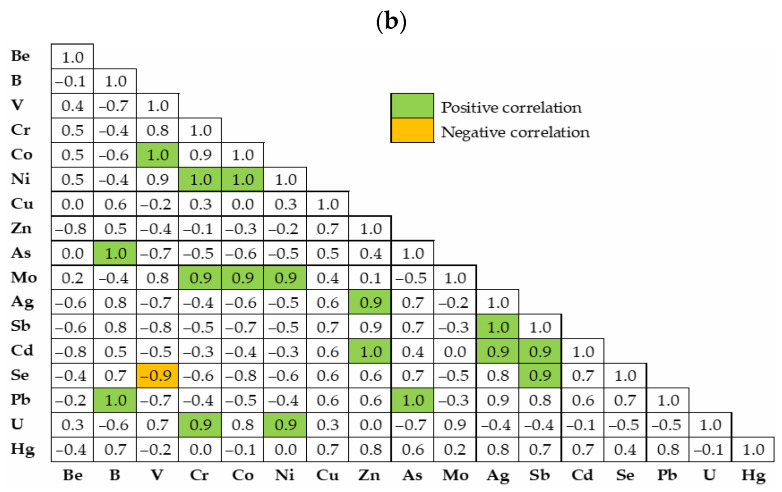
Spearman correlation matrix for studied metals and metalloids considering the soils collected from kitchen gardens in urban (**a**) and rural (**b**) areas of the northern Lisbon metropolitan area.

Strong positive correlations were found between some of the studied metals and metalloids (*p* < 0.01), as well as strong negative correlations of Se with V (for both groups) and Se with Co for urban areas (Figure 3). The relationship between these elements, as well as the significant concentrations that exceed MAV, may be due to a variety of reasons. In some cases, the geological materials present in each location may exert an influence, as is typical of volcanic rocks [54,55,64,65]. According to field observations, different remains of geological and edaphic materials from other locations (resulting from their extraction in civil work) and artefacts (e.g., building material) are used in the preparation of some kitchen gardens, being mixed with local soil. Also, the use of some organic fertilizers or manures, many of them prepared from composted sewage sludge, can increase the level of elements in soils. All these types of sources of PTEs in soils from kitchen gardens have been reported by different authors (Bidar et al. [66] and references inside). Traffic and the industrial sector can also contribute to the enrichment of certain elements in urban soils [54,55].

The distribution of the elements by different solid phases (total, pseudo-total and available) showed the existence of a variable amount associated with phyllosilicate structure (difference between total and pseudo-total concentrations) (Figure 2). These concentrations depended on elements. The evaluation of total and pseudo-total concentrations of PTE does not represent a real environmental or human risk, since only a small amount of the elements are in the available fraction, which is associated with soil solution and/or exchangeable positions associated with inorganic and organic colloids [62,67]. In general, the availability of studied elements in the soils was small, representing a small percentage of total/pseudo-total amounts. No clear relationship was obtained between available concentrations and pseudo-total and total fractions.

### 3.2. Pollution and Ecological Indexes

The indices established for determining the degree of contamination and ecological and environmental risks for different soils collected from kitchen gardens in urban and rural areas of the northern Lisbon metropolitan area were evaluated (Table 3, Tables S1 and S2).

The contamination factors (CFs) for each of the 18 PTEs included in the Portuguese soil legislation (Table S1) showed that only some soils in both groups exhibited moderate to considerable contamination for certain elements. Similar results were reported by Silva et al. [54] for some urban soils from parks and gardens. However, other urban soils were found to be highly contaminated with Cr, Ni and Pb [54].

The degree of contamination index obtained by combining these factor values (CD) varied between 3.68 and 20.07 (Table 3). The degree of contamination, as defined by Hakanson [42], enabled the identification of considerable contamination in samples UB7, UB12 and RU3, and moderate contamination in samples UB3, UB6, RU4 and RU5. This is primarily due to high levels of Ni, Co, Cr and V (Table 2 and Table S1). The same degree of contamination was identified in park soils in Lisbon (moderate and considerable), but only four PTEs were considered: Cd, Cr, Ni and Pb [54].

On the other hand, determining the modified contamination level index (mCD) with 18 elements analyzed and standardized, the level of contamination in all the soils from kitchen gardens was mild to very mild (Table 3). These results were consistent with those obtained from the contamination load index (PLI), which provides a global assessment of multi-element contamination. The PLI values for studied soils, independently of the group (urban or rural soils), was less than 1, indicating the inexistence of multi-element contamination.

At the level of ecological risk associated with each element, only moderate risks were obtained for Ni in the samples UB7 and RU3 (Table S2). Overall, none of the soils from urban or rural kitchen gardens showed a global level the ecological risk (GER) (Table 3). These results did not align with those obtained for park soils in Lisbon (e.g., ER for Cd and GER for certain park and garden areas) [54].

## 4. Conclusions

The general quality of soils in kitchen gardens in urban and rural areas of the northern Lisbon metropolitan area is limited in terms of fertility due to neutral reaction conditions, dominated by carbonate buffering, as well as unstable organic matter, favoring rapid decomposition and mineralization and loss of nutrients especially N. The availability of carbonate forms, together with the high content of available P, can limit the availability of some essential micronutrients for vegetative growth. Similarly, the cation exchange capacity, despite having high or adequate values, is dominated by Ca under conditions of Ca/Mg ratios that can limit crop growth. Thus, improving soil fertility and crop yield requires a change in soil management.

Although the pseudo-total concentrations of some elements (e.g., Cr, Ni, Cu) exceeded the maximum allowed values for agriculture use under Portuguese soil legislation, the results indicated no environmental or human risk, since the availability of the elements in simulated leachates was low. The different indices calculated (contamination indices and associated with ecological risk) also agree with this. No clear source of enrichment or elevated pseudo-total concentrations was identified in relation to the location of the kitchen gardens. However, periodic monitoring of multi-element concentrations, especially in the available fraction, is recommended to ensure environmental and human health along the time.

## Figures and Tables

**Table 1:** Main physicochemical characteristics and nutritional parameters of soils from kitchen gardens in urban and rural areas of the northern Lisbon metropolitan area (mean ± standard deviation; median). Values indicated with asterisk show significant differences between groups at *p* < 0.05.

Parameters	Unit	Urban Kitchen Garden (*n* = 15)	Rural Kitchen Garden (*n* = 5)
Coarse fraction	%	20.783 ± 9.863 22.598	23.547 ± 11.043 18.017
Fine fraction	%	79.217 ± 9.863 77.402	76.453 ± 11.043 81.983
Humidity	%	11.469 ± 10.329 8.255	22.424 ± 20.335 21.702
pH (H_2_O)	-	7.728 ± 0.406 7.770	7.504 ± 0.425 7.750
pH (KCl)	-	7.109 ± 0.324 * 7.240	6.700 ± 0.370 * 6.690
EC ^1^	µS/cm	482.347 ± 264.457 391.000	589.500 ± 412.829 497.100
Total C	g/kg	34.095 ± 19.937 28.230	47.314 ± 42.054 32.270
Organic C	g/kg	18.237 ± 13.937 16.853	30.580 ± 33.325 12.781
Labile C	g/kg	6.175 ± 1.864 5.824	8.927 ± 6.958 6.307
Total N	g/kg	2.110 ± 1.079 2.012	3.049 ± 1.762 2.024
Total S	g/kg	<2.500	<2.500
Total P	g/kg	1.065 ± 0.490 * 0.970	1.792 ± 0.974 * 1.430
Available P	mg/kg	166.794 ± 224.455 90.335	204.550 ± 180.629 267.210
CEC ^2^	cmolc/kg	44.699 ± 18.904 46.717	65.345 ± 58.749 36.013
Exchange Ca	cmolc/kg	39.327 ± 15.842 42.523	58.234 ± 56.338 28.816
Exchange Mg	cmolc/kg	3.222 ± 3.792 1.565	4.973 ± 2.827 3.925
Exchange Na	cmolc/kg	1.292 ± 0.728 1.405	0.904 ± 0.742 0.464
Exchange K	cmolc/kg	0.859 ± 0.420 0.760	1.234 ± 0.834 1.053

^1^ Electrical conductivity; ^2^ effective cation exchange capacity.

**Table 2:** Metal and metalloid concentrations in the pseudo-total fraction of soils from kitchen gardens in urban and rural areas of the northern Lisbon metropolitan area (mean (minimum and maximum)). Values indicated with asterisk show significant differences between groups at *p* < 0.05 (*) and *p* < 0.01 (**).

Element	Unit	Urban Kitchen Garden (*n* = 15)	Rural Kitchen Garden (*n* = 5)	MAV [44]
Be	mg/kg	0.820 (0.400–1.500) **	1.560 (0.900–1.900) **	2.500
B		9.300 (4.000–16.00)	14.40 (9.000–23.00)	36.00
V		58.90 (11.00–219.0)	110.8 (21.00–284.0)	86.00
Cr		53.20 (10.00–194.0)	67.80 (20.00–228.0)	67.00
Co		15.88 (2.100–63.90)	26.96 (5.500–75.80)	19.00
Ni		42.33 (5.500–191.0)	49.30 (16.40–149.0)	37.00
Cu		35.34 (11.10–92.10) *	58.82 (27.90–75.00) *	62.00
Zn		117.9 (44.00–348.0)	131.4 (77.40–237.0)	290.0
As		5.640 (1.500–11.10)	6.080 (1.900–13.20)	11.00
Mo		0.606 (0.230–1.370)	0.786 (0.230–2.050)	2.000
Ag		0.192 (0.037–1.100)	0.073 (0.049–0.120)	0.500
Sb		0.550 (0.160–1.280)	0.426 (0.130–0.880)	1.000
Cd		0.168 (0.070–0.270)	0.166 (0.100–0.340)	1.000
Se		0.470 (0.300–0.700)	0.500 (0.300–0.700)	1.200
Ta		<0.050	<0.050	1.000
Pb		29.13 (7.800–66.20)	26.3 (7.800–55.90)	45.00
U		0.890 (0.500–1.700)	0.920 (0.700–1.300)	1.900
Hg	µg/kg	73.00 (20.00–190.0)	70.00 (30.00–100.0)	160.0

**Table 3:** Results of the different indices for evaluation of environmental risk and contamination level (contamination degree indices: CD and mCD; contaminant load index: PLI and PLI zone; and the global ecological risk: GER) of the soils from kitchen gardens in urban and rural areas of the northern Lisbon metropolitan area.

	CD (*n* = 18)	mCD (*n* = 18)	PLI	PLI Zone	GER (*n* = 11)
UB1	5.34	0.30	0.15		23.23
UB2	6.42	0.36	0.19		30.61
UB3	8.31	0.46	0.23		44.47
UB4	7.39	0.41	0.24		44.15
UB5	7.02	0.39	0.23		38.03
UB6	8.19	0.46	0.26		46.66
UB7	16.60	0.92	0.27		67.25
UB8	3.68	0.20	0.12		26.65
UB9	4.54	0.25	0.15		27.36
UB10	5.09	0.28	0.17		20.06
UB11	7.72	0.43	0.22		28.00
UB12	18.35	1.02	0.40		24.24
UB13	6.62	0.37	0.20		35.09
UB14	6.29	0.35	0.20		77.24
UB15	7.64	0.42	0.22		80.01
Urban kitchen garden (*n* = 15)				0.21	
RU1	7.87	0.44	0.19		49.94
RU2	6.75	0.38	0.20		51.23
RU3	20.07	1.12	0.33		29.90
RU4	10.01	0.56	0.31		34.89
RU5	8.66	0.48	0.28		44.37
Rural kitchen garden (*n* = 5)				0.26	

## Data Availability

Data will be made available on request.

## Supplementary Materials

The following supporting information can be downloaded at https://www.mdpi.com/article/10.3390/toxics13080697/s1: Table S1: Contamination factor (CF) calculated for the soils collected from kitchen gardens in urban and rural areas of the northern Lisbon metropolitan area; Table S2: Ecological risk factors (ER), calculated for each sampling point.
