# Supplementary material for: Soil Quality and Trace Element Risk in Urban and Rural Kitchen Gardens: A Comparative Analysis
Source: Toxics. 2025 Aug 20;13(8):697. doi: 10.3390/toxics13080697 (PMC12389907; doi:10.3390/toxics13080697)
Supplement: Supplementary file 1 [file toxics-13-00697-s001.zip › toxics-3772707-supplementary.pdf]

Table S1. Degradation factor (Cf) calculated for the soils collected from kitchen gardens in urban and rural areas of the northern Lisbon metropolitan area; moderate degradation factor marked in yellow and considerable contamination marked in orange.

	Be	B	V	Cr	Co	Ni	Cu	Zn	As	Mo	Ag	Sb	Cd	Se	Ta	Pb	U	Hg
**UB1**	0.24	0.14	0.56	0.67	0.71	0.97	0.27	0.16	0.22	0.23	0.12	0.19	0.08	0.33	0.03	0.17	0.26	0.00
**UB2**	0.32	0.19	0.83	0.48	0.75	0.67	0.59	0.24	0.64	0.26	0.08	0.38	0.07	0.33	0.03	0.26	0.32	0.00
**UB3**	0.60	0.36	0.34	0.39	1.38	0.67	0.45	0.29	1.01	0.34	0.07	0.68	0.13	0.33	0.03	0.40	0.84	0.00
**UB4**	0.52	0.28	0.23	0.36	0.23	0.36	0.57	0.30	0.85	0.60	0.29	0.71	0.16	0.42	0.03	0.81	0.68	0.00
**UB5**	0.28	0.22	0.21	0.30	0.28	0.37	0.76	0.34	0.66	0.34	0.32	0.65	0.10	0.42	0.03	1.16	0.58	0.00
**UB6**	0.32	0.25	0.20	0.34	0.19	0.28	0.81	0.43	0.58	0.33	0.48	1.05	0.21	0.58	0.03	1.47	0.63	0.00
**UB7**	0.44	0.14	1.91	2.90	2.92	5.16	0.92	0.32	0.14	0.45	0.18	0.16	0.11	0.25	0.03	0.28	0.32	0.00
**UB8**	0.16	0.11	0.13	0.15	0.11	0.15	0.48	0.24	0.35	0.16	0.09	0.28	0.14	0.42	0.03	0.33	0.37	0.00
**UB9**	0.28	0.14	0.26	0.36	0.13	0.20	0.18	0.15	0.81	0.17	0.10	0.48	0.18	0.42	0.03	0.36	0.32	0.00
**UB10**	0.40	0.28	0.34	0.39	0.32	0.39	0.19	0.20	0.54	0.12	0.13	0.32	0.13	0.42	0.03	0.65	0.26	0.00
**UB11**	0.24	0.17	0.22	0.27	0.14	0.19	0.44	0.46	0.66	0.17	2.20	0.68	0.27	0.50	0.03	0.71	0.37	0.00
**UB12**	0.48	0.44	2.55	2.46	3.36	3.84	1.49	0.54	0.27	0.69	0.23	0.29	0.25	0.25	0.03	0.40	0.79	0.00
**UB13**	0.32	0.31	0.83	0.58	0.81	0.62	0.27	0.21	0.41	0.26	0.13	0.33	0.09	0.25	0.03	0.87	0.32	0.00
**UB14**	0.40	0.36	0.22	0.28	0.15	0.26	0.35	0.30	0.94	0.32	0.14	0.63	0.14	0.33	0.03	0.54	0.89	0.00
**UB15**	0.24	0.39	0.21	0.21	0.22	0.35	0.58	1.20	0.44	0.38	0.17	1.28	0.16	0.50	0.03	0.88	0.42	0.00
**RU1**	0.56	0.25	2.01	0.34	1.54	0.77	0.45	0.30	0.23	0.23	0.10	0.13	0.10	0.25	0.03	0.17	0.42	0.00
**RU2**	0.72	0.36	0.43	0.46	0.55	0.61	0.86	0.27	0.48	0.12	0.11	0.34	0.10	0.50	0.03	0.35	0.47	0.00
**RU3**	0.76	0.28	3.30	3.40	3.99	4.03	1.11	0.41	0.17	1.03	0.10	0.16	0.12	0.25	0.03	0.26	0.68	0.00
**RU4**	0.72	0.64	0.45	0.55	0.73	0.82	1.11	0.47	1.20	0.21	0.19	0.62	0.17	0.50	0.03	1.24	0.37	0.00
**RU5**	0.36	0.47	0.24	0.30	0.29	0.44	1.21	0.82	0.68	0.40	0.24	0.88	0.34	0.58	0.03	0.90	0.47	0.00

Table S2. Ecological risk factors (ER) calculated for each sampling point; moderate risk values marked in yellow.

	Cr	Co	Ni	Cu	Zn	As	Mo	Sb	Cd	Pb	Hg
**UB1**	1.34	3.55	4.86	1.34	0.16	2.18	4.05	2.47	2.40	0.87	0.01
**UB2**	0.96	3.74	3.35	2.94	0.24	6.36	4.68	4.94	2.10	1.30	0.01
**UB3**	0.78	6.92	3.35	2.27	0.29	10.09	6.03	8.84	3.90	2.00	0.01
**UB4**	0.72	1.13	1.81	2.84	0.30	8.55	10.71	9.23	4.80	4.04	0.03
**UB5**	0.60	1.42	1.84	3.79	0.34	6.64	6.12	8.45	3.00	5.80	0.03
**UB6**	0.69	0.95	1.42	4.06	0.43	5.82	5.94	13.65	6.30	7.36	0.05
**UB7**	5.79	14.61	25.81	4.58	0.32	1.36	8.01	2.08	3.30	1.38	0.02
**UB8**	0.30	0.55	0.74	2.40	0.24	3.45	2.88	3.64	4.20	1.64	0.01
**UB9**	0.72	0.66	0.99	0.90	0.15	8.09	3.06	6.24	5.40	1.79	0.01
**UB10**	0.78	1.61	1.95	0.96	0.20	5.36	2.07	4.16	3.90	3.24	0.01
**UB11**	0.54	0.71	0.96	2.21	0.46	6.64	3.06	8.84	8.10	3.56	0.02
**UB12**	4.93	16.82	19.19	7.43	0.54	2.73	12.33	3.77	7.50	1.98	0.03
**UB13**	1.16	4.05	3.09	1.36	0.21	4.09	4.59	4.29	2.70	4.33	0.01
**UB14**	0.57	0.76	1.30	1.73	0.30	9.36	5.76	8.19	4.20	2.71	0.01
**UB15**	0.42	1.08	1.76	2.88	1.20	4.36	6.84	16.64	4.80	4.38	0.02
**RU1**	0.69	7.68	3.84	2.25	0.30	2.27	4.05	1.69	3.00	0.87	0.01
**RU2**	0.93	2.76	3.03	4.31	0.27	4.82	2.07	4.42	3.00	1.76	0.01
**RU3**	6.81	19.95	20.14	5.56	0.41	1.73	18.45	2.08	3.60	1.28	0.02
**RU4**	1.10	3.63	4.09	5.55	0.47	12.00	3.69	8.06	5.10	6.21	0.03
**RU5**	0.60	1.45	2.22	6.05	0.82	6.82	7.11	11.44	10.20	4.51	0.03
